# A guinea pig model of Zika virus infection

**DOI:** 10.1186/s12985-017-0750-4

**Published:** 2017-04-11

**Authors:** Mukesh Kumar, Keeton K. Krause, Francine Azouz, Eileen Nakano, Vivek R. Nerurkar

**Affiliations:** Department of Tropical Medicine, Medical Microbiology and Pharmacology, Pacific Center for Emerging Infectious Diseases Research, John A. Burns School of Medicine, University of Hawaii at Manoa, Honolulu, 651 Ilalo Street, BSB 320, Honolulu, HI 96813 USA

**Keywords:** Zika virus, Microcephaly, Guinea pigs, Animal models, Flavivirus, Immune response, Pathogenesis

## Abstract

**Background:**

Animal models are critical to understand disease and to develop countermeasures for the ongoing epidemic of Zika virus (ZIKV). Here we report that immunocompetent guinea pigs are susceptible to infection by a contemporary American strain of ZIKV.

**Methods:**

Dunkin-Hartley guinea pigs were inoculated with 10^6^ plaque-forming units of ZIKV via subcutaneous route and clinical signs were observed. Viremia, viral load in the tissues, anti-ZIKV neutralizing antibody titer, and protein levels of multiple cytokine and chemokines were analyzed using qRT-PCR, plaque assay, plaque reduction neutralization test (PRNT) and multiplex immunoassay.

**Results:**

Upon subcutaneous inoculation with PRVABC59 strain of ZIKV, guinea pigs demonstrated clinical signs of infection characterized by fever, lethargy, hunched back, ruffled fur, and decrease in mobility. ZIKV was detected in the whole blood and serum using qRT-PCR and plaque assay. Anti-ZIKV neutralizing antibody was detected in the infected animals using PRNT. ZIKV infection resulted in a dramatic increase in protein levels of multiple cytokines, chemokines and growth factors in the serum. ZIKV replication was observed in spleen and brain, with the highest viral load in the brain. This data demonstrate that after subcutaneous inoculation, the contemporary ZIKV strain is neurotropic in guinea pigs.

**Conclusion:**

The guinea pig model described here recapitulates various clinical features and viral kinetics observed in ZIKV-infected patients, and therefore may serve as a model to study ZIKV pathogenesis, including pregnancy outcomes and for evaluation of vaccines and therapeutics.

## Background

Zika virus (ZIKV) is an emerging mosquito-borne pathogen that is part of the Spondweni serocomplex of the genus *Flavivirus*, family *Flaviviridae*. The ZIKV genome is comprised of a single-stranded, positive-sense 11-kb RNA that contains three structural (capsid, pre-membrane, and envelope) and seven nonstructural (NS1, NS2A, NS2B, NS3, NS4A, NS4B, and NS5) genes [1]. Little is known about ZIKV pathogenesis, however it is thought that after an infected mosquito bite, viral replication occurs in local dendritic cells with subsequent spread to lymph nodes and the bloodstream. Viremia is generally seen within 3 to 4 days after onset of symptoms. Approximately 80% of individuals infected with ZIKV have no symptoms. Patients with symptomatic ZIKV infection usually present with a mild febrile illness characterized by fever, rash, arthralgia, myalgia, headache, and conjunctivitis [1, 2]. During the first week after onset of symptoms, ZIKV infection can often be diagnosed by performing qRT-PCR on serum specimens. ZIKV-specific IgM and neutralizing antibodies typically develop toward the end of the first week of illness [1].

ZIKV outbreaks have been recorded in the United States and Hawaii has encountered few cases of travel related ZIKV [3–5]. Using archived blood samples, we recently demonstrated ZIKV IgM and IgG antibodies in six mothers who gave birth to babies with microcephaly in Hawaii between 2009 and 2012 [6]. During the current epidemic in Latin America, ZIKV infection has been linked to the development of severe fetal abnormalities that include spontaneous abortion, stillbirth, hydranencephaly, microcephaly, and placental insufficiency that may cause intrauterine growth restriction [1, 7]. We recently reported the first congenital ZIKV infected case in the United States, confirmed by high ZIKV IgM antibody titers in serum and cerebrospinal fluid [5]. The reported congenital malformation cases may represent only the most severe form of the disease, with less severe infection producing long-term cognitive or functional sequelae. No effective therapies currently exist for treating patients infected with ZIKV.

Small animal models of ZIKV pathogenesis are a key research priority in response to the current ZIKV epidemic. Recently, ZIKV infection and disease have been evaluated in non-pregnant and pregnant wild-type mice, as well as a large panel of immunodeficient transgenic mice [8–10]. It has been demonstrated that ZIKV infection does not cause disease in weaned wild-type mice [8, 9]. Young A129 mice (lacking interferon α/β receptor) and AG129 (lacking interferon α/β and γ receptors) were recently reported to succumb to ZIKV infection and to develop neurological signs [8, 10, 11]. Since these mouse models are deficient in innate immune response, an immunocompetent animal model is warranted. Initial experiments conducted in 1950s showed that guinea pigs inoculated intracerebrally with the African ZIKV strain MR 766 developed no signs of infection [12]. These studies used the prototype MR 766 strain of ZIKV, which had undergone extensive passage in suckling mouse brains, and to date no experiments have been reported in guinea pigs with more contemporary ZIKV isolates of greater clinical relevance [12]. Molecular studies of the ZIKV strains currently circulating in South and Central America have revealed that the virus originated from the Asian lineage of the virus [13]. These viruses are genetically distinct from the African lineage of the virus [13].

The guinea pig is a well-accepted model for studies on congenital infections and sexual transmission [14, 15]. The guinea pig model is physiologically and immunologically more similar to humans than other small animal models [15]. Specifically, the guinea pig’s reproductive physiology and estrous cycle are similar to humans. Also, placentation in the guinea pig occurs in a manner similar to that of humans, and both guinea pig and human placentas are classified as hemomonochorial [14–16]. Similar to humans, the placenta of guinea pigs has a single trophoblast layer that separates maternal and fetal circulations. Guinea pigs have a long gestation period and pups are born with mature central nervous system (CNS) [15], which makes this species a promising subject for studies of *in utero* transfer of ZIKV and neurological manifestations in infants. Based on the aforementioned positive traits of the guinea pig model, we evaluated ZIKV infection in guinea pigs using a contemporary ZIKV strain, PRVABC59 (PR 2015), isolated from serum of an infected patient in Puerto Rico in December 2015 [13, 17].

## Methods

### Ethics statement

This study was conducted according to the NIH Guide for the Care and Use of Laboratory Animals after approval of the University of Hawaii’s (UH) Institutional Animal Care and Use Committee (IACUC) (Protocol number: 16-2377). Guinea pigs were housed in groups and appropriate environmental enrichment was provided. Food and sterile water were available *ad libitum*. Animals infected with ZIKV were housed in seal-safe cages under climate-control conditions. All animal experiments were conducted in the certified isolated animal biosafety level-2 (ABSL-2) suite in consultation with veterinary and animal care staff at the UH. For animal procedures, guinea pigs were anaesthetized with 1.5 to 2% isofluorane in oxygen in an induction chamber until full sedation was achieved.

### ZIKV infection experiments

Dunkin-Hartley guinea pigs of both sexes weighing 200–250 g (Charles River Laboratories) were used for animal infection studies. All virus- culture and -infection experiments were conducted after approval from the UH Institutional Biosafety Committee (Protocol number: 16-12-529-01-1A) in the certified isolated biosafety level-2 (BSL-2) suite. We used a sequence-verified ZIKV strain, PRVABC59 (PR 2015), isolated from the serum of an infected patient in Puerto Rico in December 2015 (BEI Resources, NR-50240). Virus strain was amplified once in Vero E6 cells (ATCC) and had titers of 2X10^6^ plaque-forming units (PFU)/mL. Virus was grown in M199 media (GIBCO) containing 5% fetal bovine serum. After acclimatization for 5 days, nine animals were inoculated with 10^6^ PFU of ZIKV via subcutaneous route. Clinical signs and mortality were observed in animals as described previously in mouse and guinea pig models of viral infections [18–20]. Briefly, clinical signs were measured daily and a numerical value was assigned to a group of clinical signs and recorded to aid analysis. Animals were monitored using a morbidity scale as follows: 1, healthy; 2, mild signs of lethargy; 3, mild signs of lethargy, ruffled fur, hunched posture; 4, increased lethargy, limited mobility, ruffled fur, hunched posture; 5, moribund with minimal mobility and inability to eat food or drink water. Animals were uniquely identified, weighed and rectal temperature was recorded daily.

On days 2, 3, and 5 after inoculation, groups of three animals were euthanized and blood samples were obtained by cardiac puncture. Blood was allowed to clot at room temperature and serum was separated by centrifugation. One hundred μL of whole blood was also collected in RNAprotect Animal Blood Tubes (Qiagen) for extracting RNA from blood. Animals were perfused with 20 mL of cold PBS. Spleen and brain were harvested and flash frozen in 2-methylbutane as described previously [19, 21, 22]. Similarly, blood and tissues were obtained from four mock-infected guinea pigs. All tissues, whole blood and serum from infected animals were stored at −80 °C until virus titration.

### qRT-PCR

RNA from tissue samples, serum and urine from ZIKV-infected and mock-infected animals was extracted with the RNeasy Mini Kit (tissues) or Viral RNA Mini Kit (serum and urine) (Qiagen) as described previously [21, 23, 24]. RNA from whole blood was extracted using RNeasy Protect Animal Blood Kit (Qiagen). A previously published primer set was used to detect ZIKV RNA: forward, 5′-CCGCTGCCCAACACAAG-3′; reverse, 5′-CCACTAACGTTCTTTTGCAGACAT-3′; probe, 5′-/56-FAM/AGCCTACCT/ZEN/TGACAAGCAATCAGACACTCAA/3IABkFQ/-3′ (Integrated DNA Technologies) [25]. Cycling conditions were as follows: 95 °C for 15 min, followed by 40 cycles of 94 °C for 15 s and 60 °C for 60 s. Viral burden was expressed on a log_10_ scale in serum and blood as genome copies per milliliter, and viral burden in tissues as genome copies per μg tissue RNA after comparison with a standard curve produced using serial 10-fold dilutions of ZIKV RNA [23].

### Plaque assay

For plaque assay, 100 μL of undiluted serum was added to a confluent monolayer of Vero cells in 6-well plates and incubated for 1 hr at 37 °C and 5% CO_2_ as described previously [18, 19]. Vero cells were overlaid with 3 mL of M-199 medium containing 1% agarose. Three days later, a second overlay of 3 mL M-199 medium containing 1% agarose and 1% neutral red was added for plaque visualization. The plaques were scored on day 2 after second overlay as described previously [19]. Since only up to 10 plaques were observed after incubation with undiluted serum, further dilutions of serum were not conducted.

### Plaque reduction neutralization test (PRNT)

The titer of anti-ZIKV neutralizing antibody was measured in the serum using PRNT assay as described previously [26, 27]. Serum collected from ZIKV-infected and mock-infected animals were serially diluted from 1:20 to 1:1280, and PRNT was conducted against ZIKV PRVABC59 strain. The highest dilution of serum resulting in 50% reduction in the number of plaques compared to the growth of the virus control was determined as described previously [26].

### Measurement of cytokines and chemokines

The levels of cytokines and chemokines were measured in the serum collected from ZIKV-infected and mock-infected animals by multiplex immunoassay using MILLIPLEX MAP Rat Cytokine/Chemokine magnetic panel as per manufacturer’s instructions (Millipore) [19, 23]. We analyzed protein levels of IL-2, IL-5, IL-18, IL-12 (p70), IL1-α, IL-1b, IL-17A, IL-4, IL-6, IL-13, IL-10, TNF-α, G-CSF, MCP-1, MIP-1α, LIX, Fractalkine, VEGF, RANTES, GM-CSF, Eotaxin, MIP2, Leptin, EGF, IFNγ, KC and IP-10. Species cross reactivity was evaluated by the manufacturer (http://www.abacus-als.com/media/Milliplex_2014.pdf).

### Data analysis

Mann-Whitney test using GraphPad Prism 5.0 (GraphPad Software, La Jolla, California, United States) was used to calculate P values of difference. Data are represented as the mean ± standard error of the mean (SEM). Differences of *p* < 0.05 were considered significant.

## Results and discussion

### Guinea pigs demonstrate clinical signs of infection

**Fig. 1:**
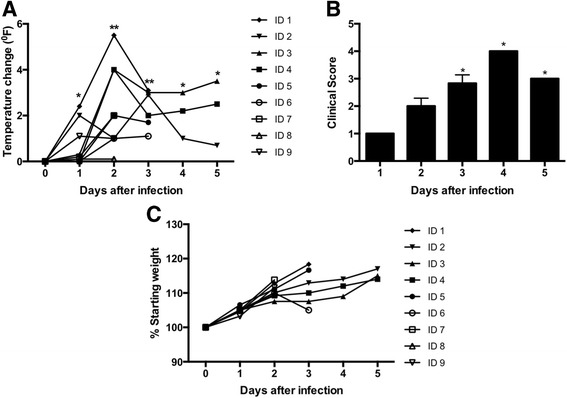
Clinical signs of ZIKV-infected guinea pigs: **a** Temperature difference in animals compared with baseline taken on the day of infection. *p < 0.05, **p < 0.001. **b** Clinical score of animals after infection. The designation for the clinical scores is as follows: 1, healthy; 2, mild signs of lethargy; 3, mild signs of lethargy, fur ruffling, hunched posture; 4, increased lethargy, limited mobility, ruffled fur, hunched posture; 5, moribund with minimal mobility and inability to eat food or drink water (*n* = 3 –9 per time point). *p < 0.05 (compared to the baseline taken on the day of infection). **c** Weight change depicted as percentage difference compared to the day of infection

In this study, to mimic the natural mosquito-bite route of ZIKV transmission, nine guinea pigs were inoculated subcutaneously with ZIKV. Based on recent ZIKV studies using mouse models and non-human primates, we inoculated guinea pigs with 10^6^ PFU of ZIKV [8, 9, 28]. Previous studies with related flaviviruses such as West Nile virus (WNV) and dengue virus also estimated that mosquitoes deliver 1X10^4^ to 1X10^6^ PFU of virus [29, 30]. This is also the range found in mosquito saliva in a recent publication specifically evaluating Brazilian ZIKV [31]. In this study, we used a low cell-culture-passaged and sequence-verified ZIKV strain, PRVABC59 (PR 2015), isolated from the serum of an infected patient in Puerto Rico in December 2015. This strain is closely related to the epidemic strains circulating in the Americas that have been linked to *in utero* ZIKV infection [13, 17]. Upon ZIKV infection, animals demonstrated clinical signs of infection characterized by fever, lethargy, hunched back, ruffled fur, and decrease in mobility. Within 2 days after infection, eight out of nine animals displayed fever (1°-5° F increase in rectal temperature). At day 3 after infection, all remaining six animals displayed fever (1°-3° F increase in rectal temperature). Two out of three remaining animals continued to display mild fever till day 5 after infection (Fig. 1a). Infected animals demonstrated signs of illness characterized by lethargy, hunched posture, ruffled fur, and decrease in mobility (Fig. 1b). Similar to our data, it has been demonstrated that subcutaneous inoculation of ZIKV causes fever in rhesus macaques [32]. In this study guinea pigs did not loose any significant weight (Fig. 1c). Also, none of the infected guinea pigs met humane endpoints or died. One limitation of this study was that animals were only monitored for five-days after infection, and further studies are warranted to look into long-term ZIKV disease progression in guinea pigs.

### ZIKV causes viremia in guinea pigs

**Fig. 2:**
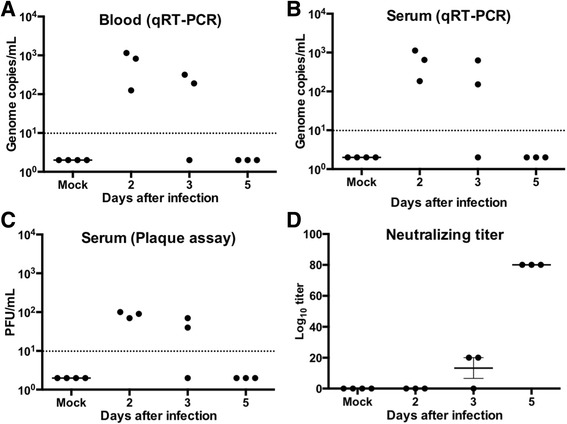
ZIKV load in guinea pig serum: ZIKV RNA loads measured in **a** whole blood and **b** serum at days 2, 3, and 5 after infection. **c** Number of PFU/mL of ZIKV in serum of animals. Each data point represents an individual guinea pig (*n* = 3 –4 per group). Data points below the horizontal line are below the limit of detection of the assay. **d** PRNT was conducted using serum collected from ZIKV-infected and mock-infected animals. Data are expressed as mean log_10_ titer ± SEM (*n* = 3-4 per group)

Viremia is well documented as the marker for flavivirus replication in vivo. Blood was collected from groups of three animals on days 2, 3 and 5 after infection. ZIKV RNA was detected in serum and whole blood by ZIKV-specific qRT-PCR in all three animals at day 2 after infection, and two out of three animals at day 3 after infection. By day 5 after infection, viremia was undetectable in all remaining animals (Fig. 2a and b). Levels of ZIKV RNA detected in the serum and whole blood were similar. We also conducted plaque assays to measure infectious ZIKV in the serum. Consistent with the qRT-PCR results, we detected infectious ZIKV in all animals at day 2 after infection, and two out of three animals at day 3 after infection (Fig. 2c). No infectious virus was detected in all animals euthanized at day 5 after infection. ZIKV was not detected in the serum of mock-infected animals. The short timeframe of detectable ZIKV RNA compares with observations in humans with transient viremia even during the acute phase of clinical disease [1, 25].

We next measured anti-ZIKV neutralizing antibody titer in the serum of infected animals using plaque reduction neutralization test (PRNT). Anti-ZIKV neutralizing antibody was not detected in the serum of all four mock-infected animals. Anti-ZIKV neutralizing antibody was not detected at day 2 after infection. Low anti-ZIKV neutralizing antibody titer (1:20) was detected in two out of three animals at day 3 after infection. Neutralizing antibody titer increased further at day 5 after infection (1:80), and was detected in all three animals (Fig. 2d). Overall, neutralizing antibody titer was low in all the animals. Further studies using larger cohort of infected and un-infected guinea pigs, and additional time points are warranted to further confirm neutralizing antibody titers. Together, these results demonstrate active ZIKV replication in guinea pigs.

### ZIKV induces multiple cytokines and chemokines in serum

**Fig 3:**
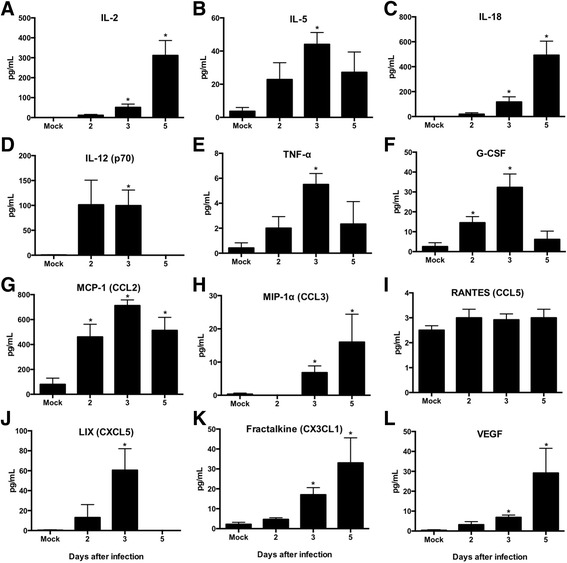
Cytokines, chemokines and growth factors levels in serum of ZIKV-infected guinea pigs: Serum was collected from mock and ZIKV-infected guinea pigs at indicated days after infection. Levels of cytokines, chemokines and growth factors as noted in the figure (**a**-**l**) were measured using multiplex immunoassay, and are expressed as the mean concentration (pg/mL) ± SEM (*n* = 3 –4 per group). *p < 0.05 (compared to mock-infected)

Increased production of cytokines and chemokines is a common event observed during infection with flavivirus such as WNV, Japanese encephalitis virus (JEV) and dengue virus [19, 23, 33]. These flavivirus-induced cytokines and chemokines play an important role in the modulation of host defense and disease manifestation [33–36]. To date, no studies have examined the inflammatory response in ZIKV-infected animals. Therefore, we next assessed the protein levels of multiple cytokines, chemokines and growth factors in the serum of mock-infected and ZIKV-infected guinea pigs using multiplex immunoassay [19, 23]. Very low levels of these cytokines, chemokines and growth factors were detected in the serum of mock-infected guinea pigs (*n* = 4) (Fig. 3). We observed a slight increase in the levels of IL-5, IL-12 (p70), G-CSF and MCP-1 in ZIKV-infected guinea pigs at day 2 after infection. ZIKV infection resulted in a significant increase in protein levels of IL-2, IL-5, IL-18, IL-12 (p70), TNF-α, G-CSF, MCP-1, MIP-1α, LIX, Fractalkine and VEGF at day 3 after infection (Fig. 3). While levels of IL-2, IL-18, MIP-1α, CX3CL1 and VEGF further increased at day 5 after infection, levels of IL-5, IL-12 (p70), TNF-α, G-CSF, MCP-1, and LIX decreased at day 5 after infection. Highest induction was observed in the levels of IL-2, IL-18 and MCP-1. Levels of RANTES did not differ between mock and ZIKV-infected guinea pigs. The increased production of inflammatory cytokines observed in ZIKV-infected guinea pigs could have multiple effects including enhanced virus replication and virus entry into the brain.

Induction of IL-18 is particularly of interest. IL-18 is a pleiotropic cytokine involved in the regulation of innate and acquired immune response [37]. IL-18 has been shown to play a protective role in several viral infections including vaccinia virus, influenza virus, and herpes simplex virus-1 [38], and is induced following infection with JEV and hepatitis C virus [33, 39]. IL-18 also promotes production of various cytokines by CD4^+^ T helper cells [37]. Conversely, previous studies have implicated IL-18 in the pathogenesis of various diseases including autoimmune diseases, myocardial function, emphysema, metabolic syndromes, psoriasis, inflammatory bowel disease, macrophage activation syndrome, sepsis and acute kidney injury [37], and may play a pathological role in recurrent pregnancy loss [40]. Epidemiological studies indicate that optimal IL-18 levels are critical for successful pregnancy [41]. Therefore up-regulation of IL-18 may have significant implications on pregnancy during ZIKV infection. Similar to IL-18, IL-2 can induce the production of other cytokines, including TNF-α and granulocyte-macrophage colony-stimulating factor. IL-2 also stimulates the proliferation of CD4^+^ and CD8^+^ T cells and NK cells, and enhances cytolytic activity against a variety of target cells [42]. The elevated cytokine and chemokine levels observed in this study warrant further research to determine their role in ZIKV infection and pathology.

### ZIKV infects guinea pig brain

**Fig. 4:**
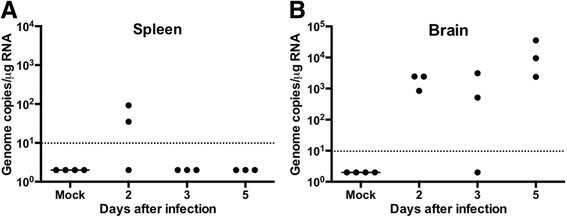
ZIKV load in guinea pig tissues: ZIKV RNA load was measured in **a** spleen and **b** brain at days 2, 3, and 5 after infection. Each data point represents an individual guinea pig (*n* = 3 –4 per group). Data points below the horizontal line are below the limit of detection of the assay

We also measured ZIKV RNA by qRT–PCR in spleen and brain. Cardiac perfusion with cold PBS was conducted in these animals to reduce peripheral blood contamination. ZIKV was detected in the spleen of two out of three animals at day 2 after infection (Fig. 4a). No virus was detected in the spleen at day 3 and 5 after infection. Interestingly, high ZIKV RNA was detected in the brains of eight out of nine animals, with highest ZIKV load detected at day 5 after infection (Fig. 4e). It is interesting to note that virus in the blood was undetectable in all animals at day 5 after infection (Fig. 2a, b, c).

The neurotropic nature of ZIKV has been observed in various animal models and in humans [2, 9, 12, 32, 43]. Our data demonstrate that the contemporary ZIKV strain can invade and replicate within the brain of guinea pigs after subcutaneous inoculation. Previous reports show that in immunocompromised mice, A129, AG129, with defective IFN responses, high ZIKV load was detected in the brain and spinal cord [9, 11]. Similarly, ZIKV RNA has also been detected in non-human primates infected subcutaneously with ZIKV [28, 32]. While much remains to be determined about the mechanisms by which ZIKV mediates microcephaly, brain and the nervous system are relevant to aspects of ZIKV disease in humans including Guillain-Barré syndrome, congenital infection, and microcephaly.

### Guinea pig as a model for Zika virus infection

The mechanism of ZIKV pathogenesis remains largely unclear. This is partly due to the lack of a robust animal model that recapitulates the clinical manifestations and disease kinetics as observed in ZIKV-infected patients. Our results demonstrate that guinea pigs can be infected with the contemporary ZIKV strain circulating in Americas. Guinea pigs have been previously demonstrated to be infected with other flaviviruses including tick-borne encephalitis virus, JEV and WNV [44–46]. The guinea pig model offers unique advantages for the study of vaccines against transplacental infections. This feature of guinea pig biology is exploited for studies focused on transplacental pathogens such as cytomegalovirus (CMV), syphilis and *Listeria monocytogenes* [14, 47, 48]. It has been shown that pregnant guinea pigs are substantially immunocompromised compared to non-pregnant animals with respect to CMV infection [49]. Therefore, enhanced ZIKV disease with broad tissue tropism may be present in pregnant guinea pigs.

## Conclusions

This was a proof-of-concept study designed to establish the infectivity and viral dynamics of contemporary ZIKV infection in guinea pigs. In this study we demonstrate for the first time that ZIKV induces production of potent cytokines, chemokines and growth factors in the serum of infected animals. The guinea pig model described in this study recapitulates various clinical features and viral kinetics observed in ZIKV-infected patients, and therefore may serve as a model to study ZIKV pathogenesis, including pregnancy outcomes and for evaluation of vaccines and therapeutics. Future studies using pregnant guinea pigs, alternative routes of infections, and other ZIKV strains including guinea pig adapted ZIKV strains are warranted to further refine this newly developed guinea pig model.

## Abbreviation

JEV: Japanese encephalitis virus
PFU: Plaque-forming units
PRNT: Plaque reduction neutralization test
qRT-PCR: Quantitative, real-time PCR
WNV: West Nile virus
ZIKV: Zika virus
